# Supplementary material for: Tissue and cellular spatiotemporal dynamics in colon aging
Source: bioRxiv. 2024 Apr 26:2024.04.22.590125. Preprint. [Version 1] doi: 10.1101/2024.04.22.590125 (PMC11071407; doi:10.1101/2024.04.22.590125)

Extended Figure 1

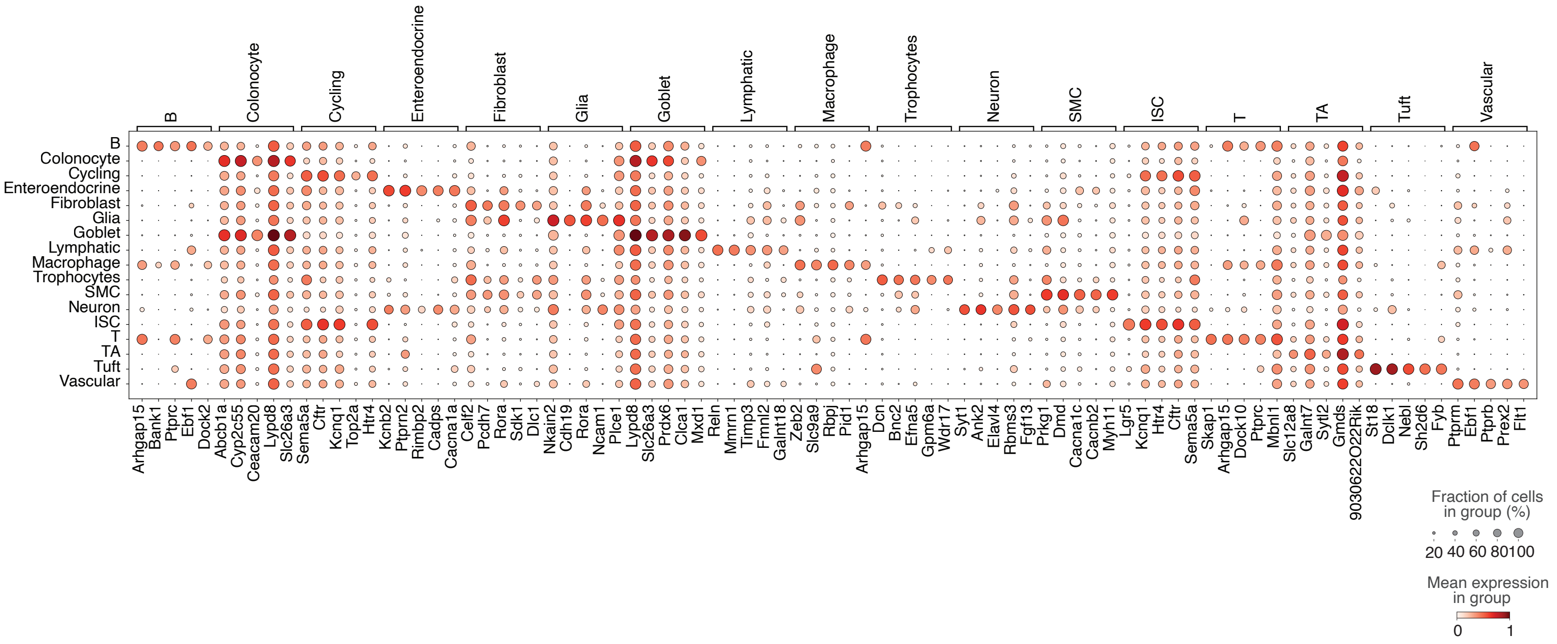

Extended Figure 2

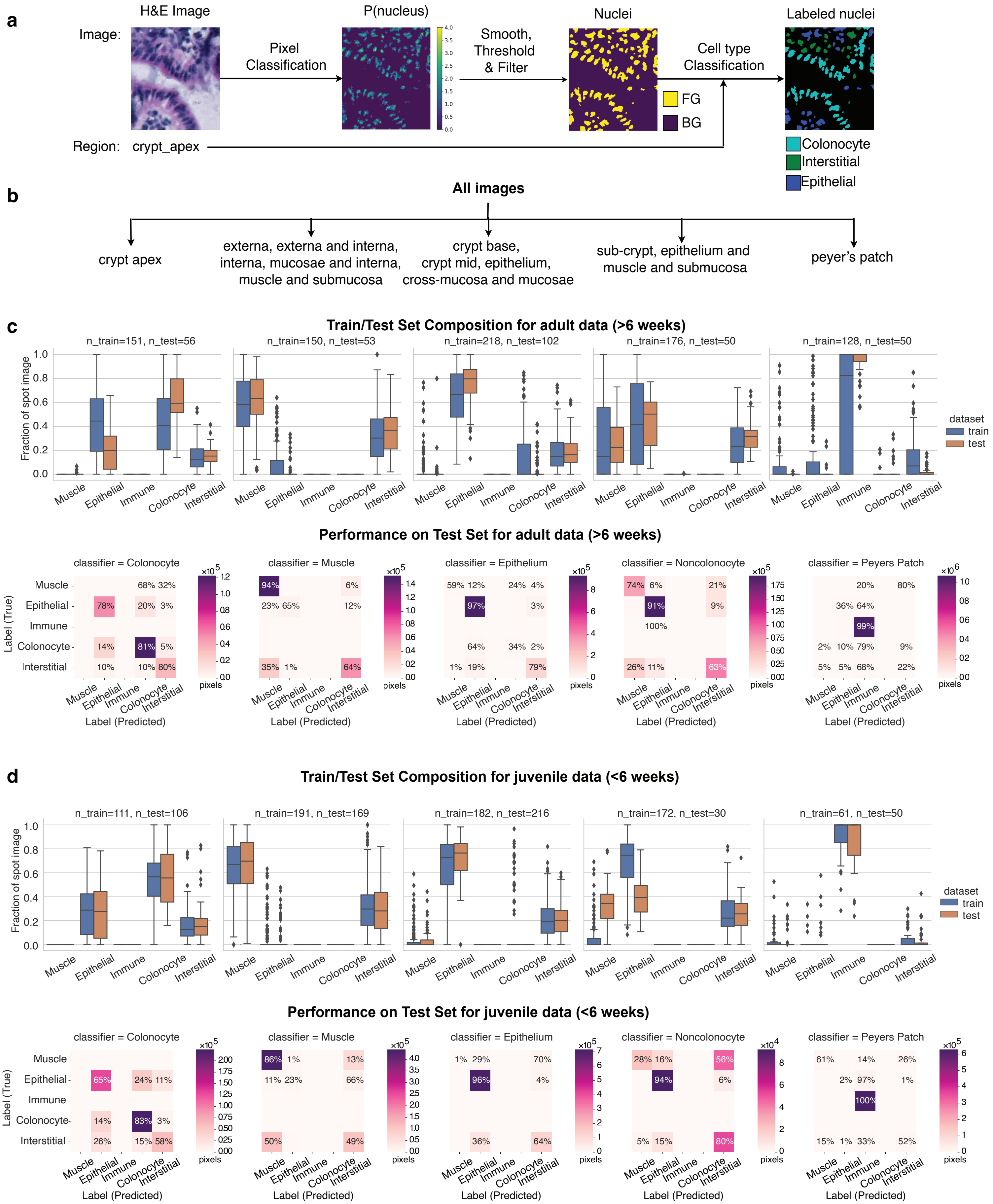

Extended Figure 3

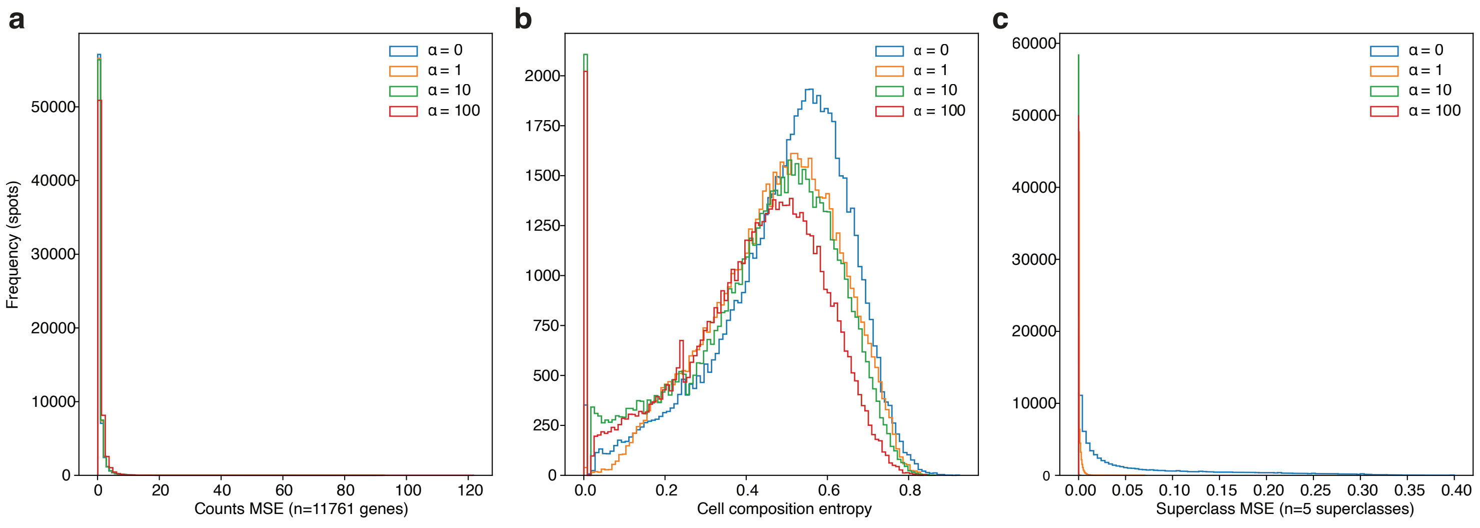

Extended Figure 4

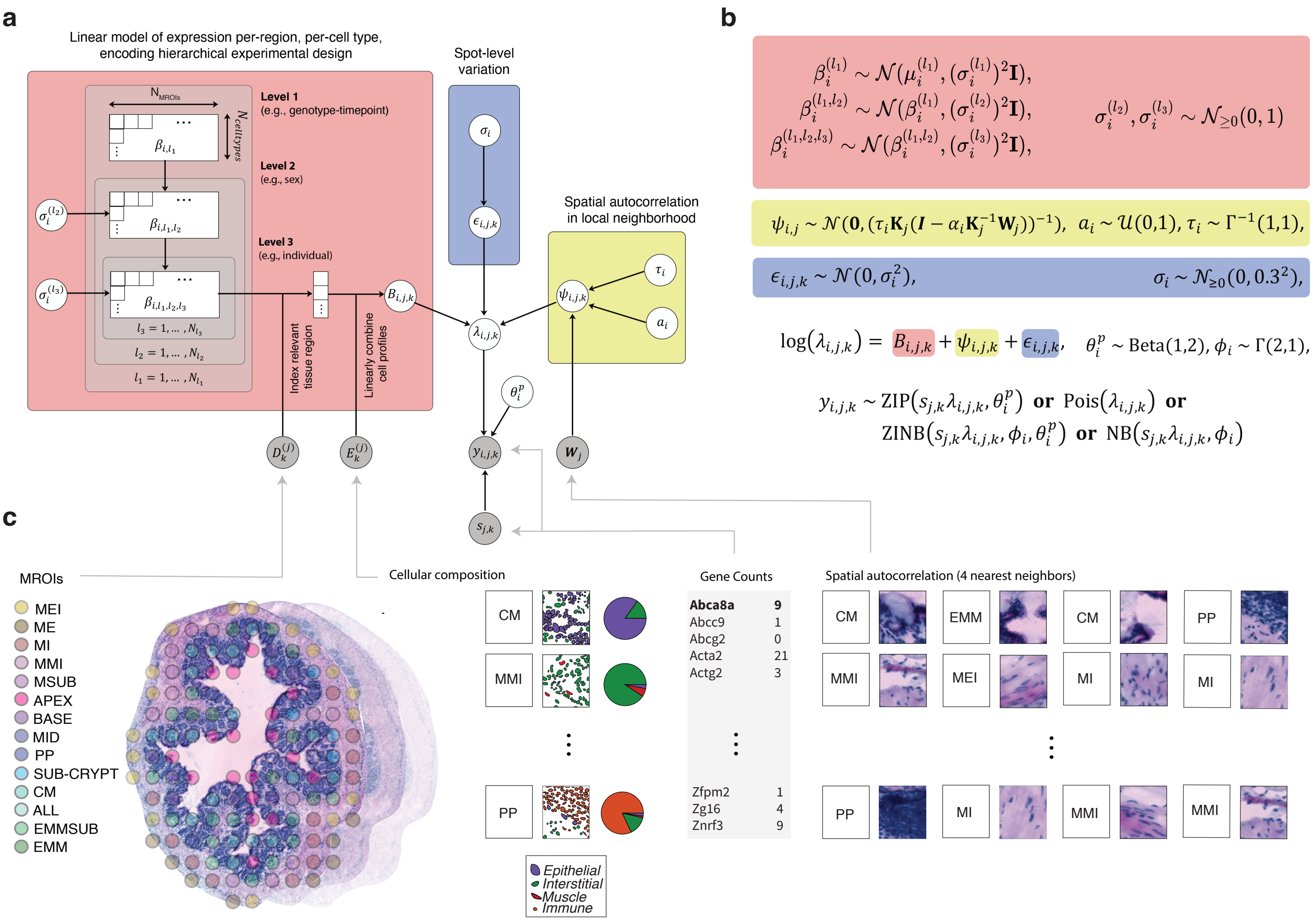

Extended Figure 5

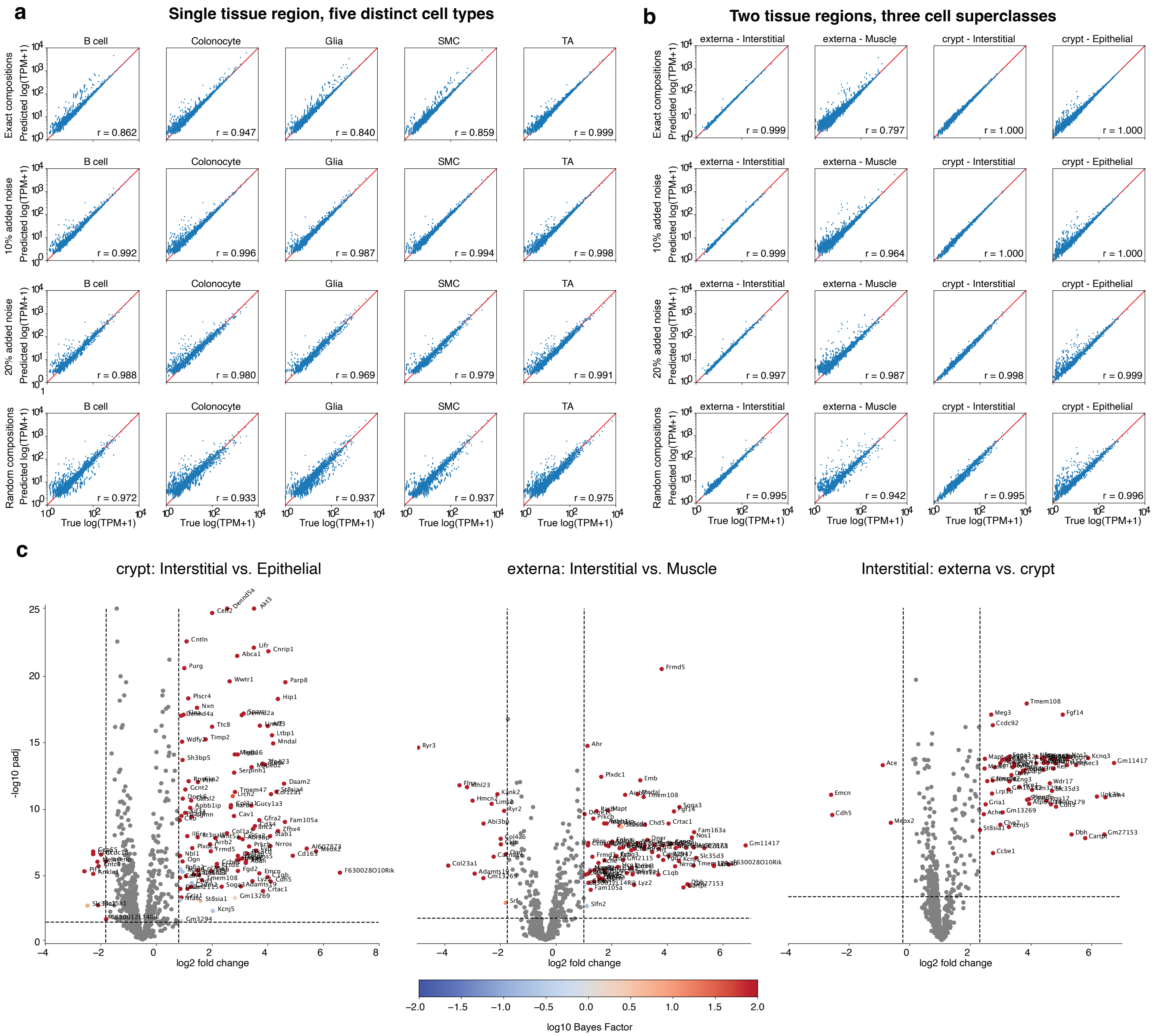

Extended Figure 6

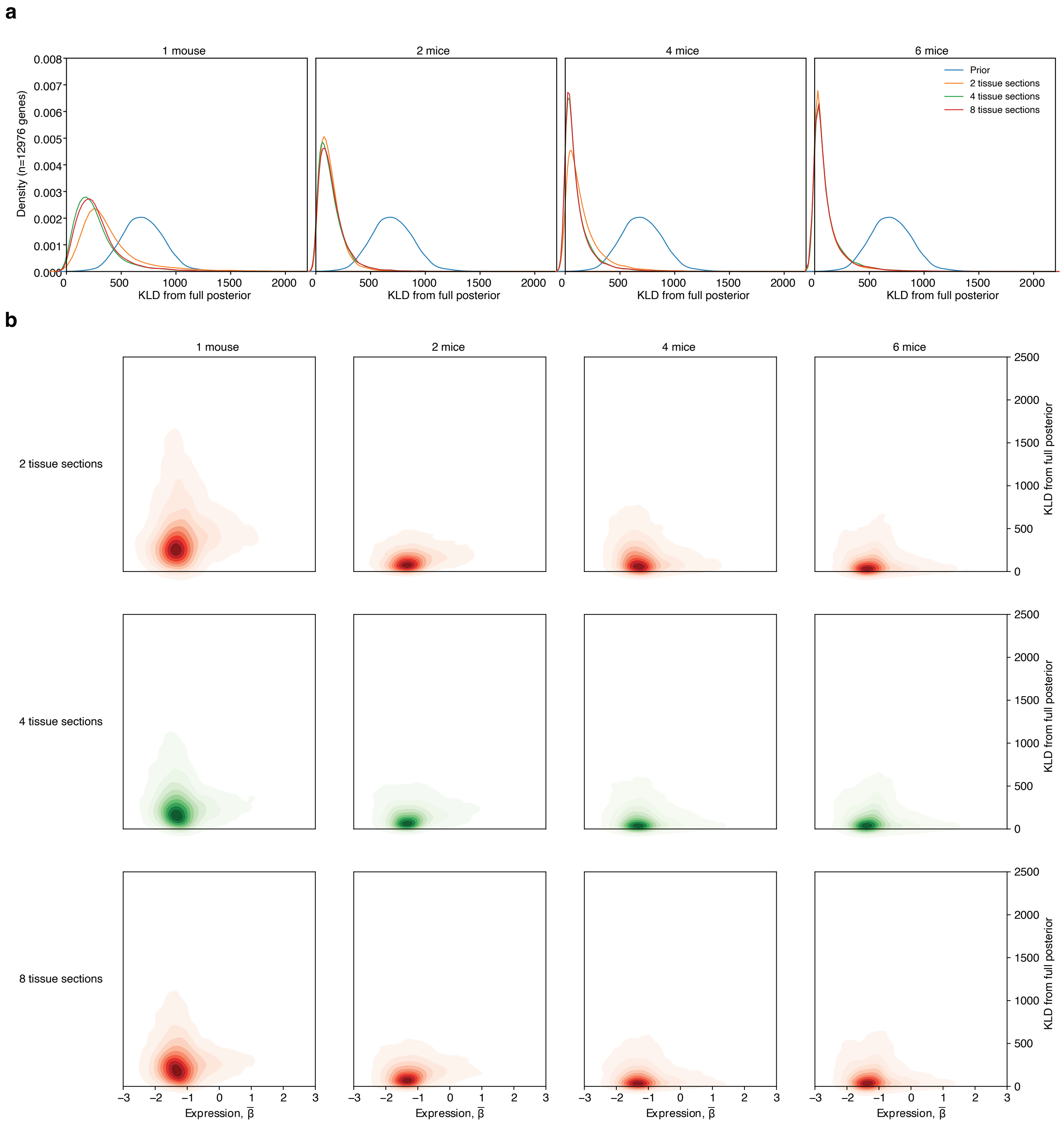

Extended Figure 7

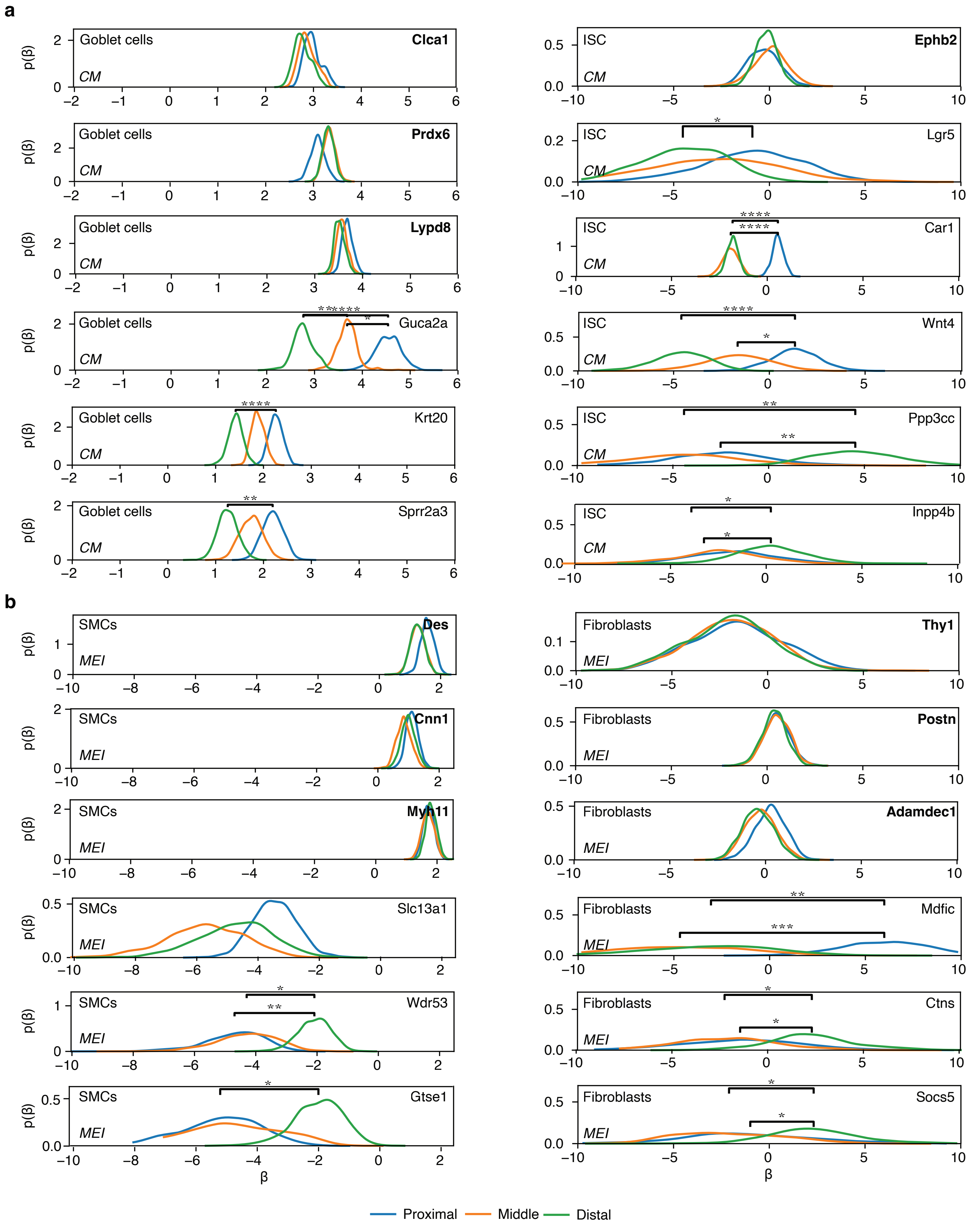

Extended Figure 8

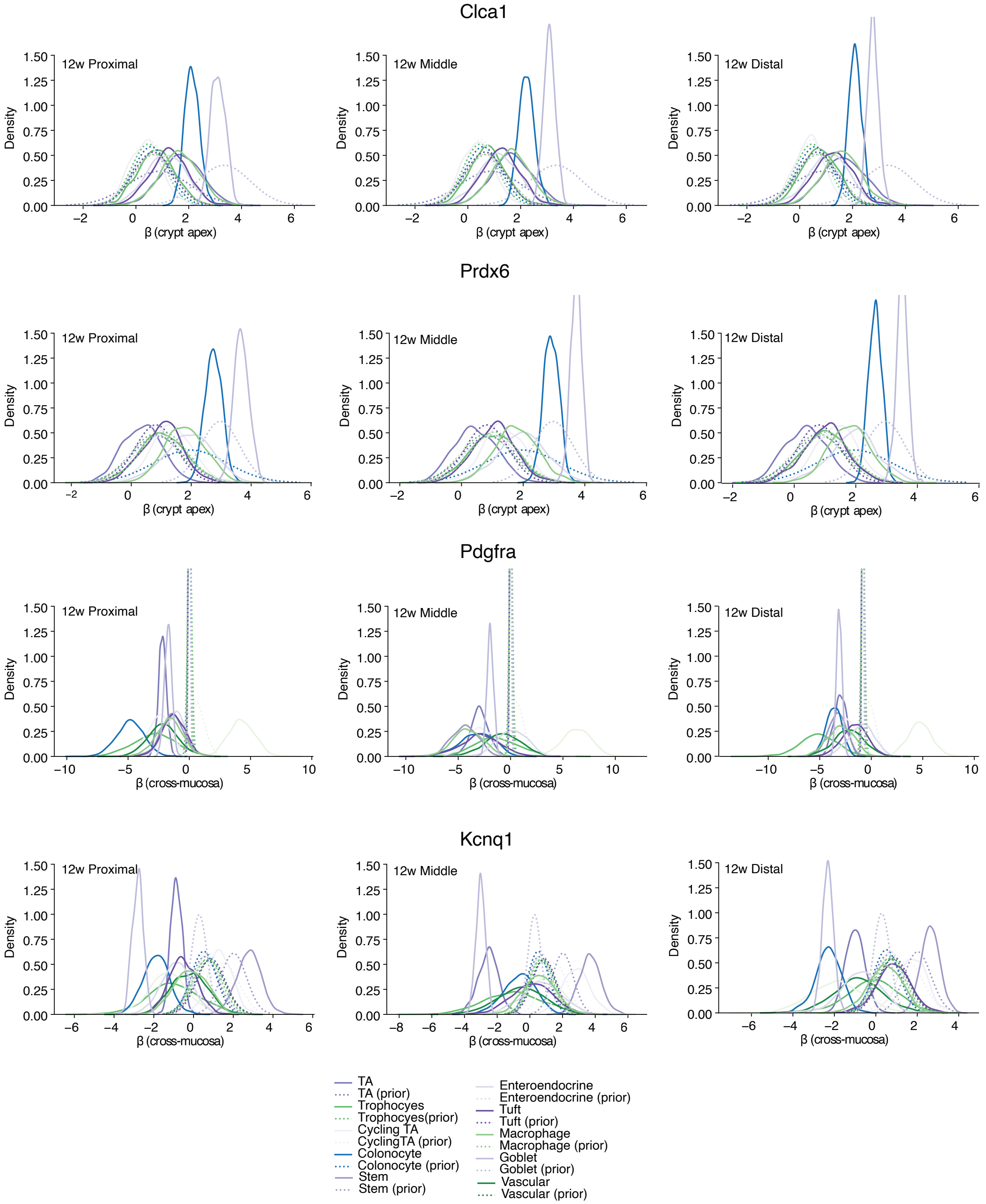

Extended Figure 9

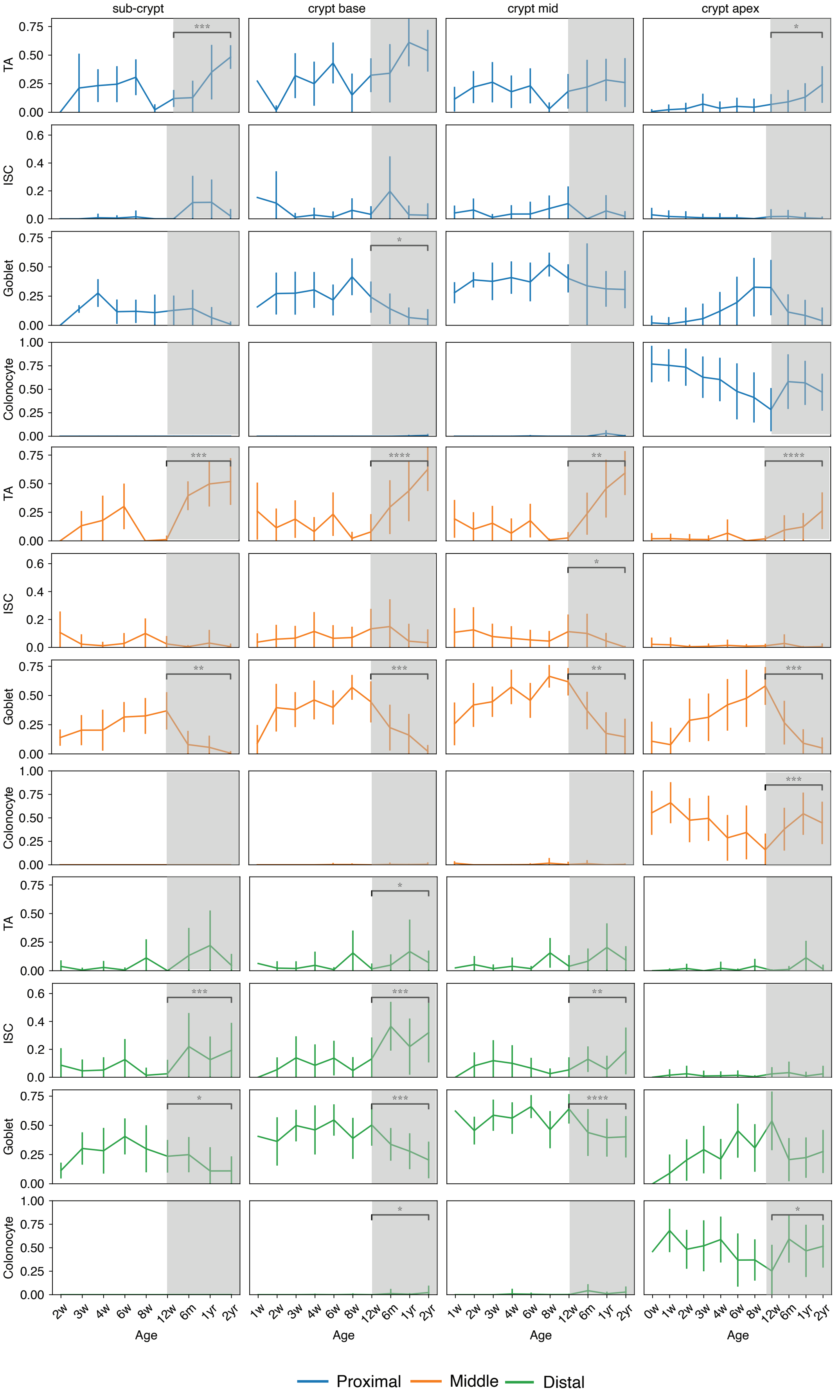

Supplement: Supplement 1 — Extended Data Fig. 1: Cell type associated genes from snRNA-seq. Mean expression (dot size) and fraction of expression cells (dot color) of the top three marker genes (columns) for each of the 17 cell subsets (columns) defined from snRNA-seq. Extended Data Fig. 2: Validation of semantic segmentation of morphological cell types (“super-classes”). (a,b) Semantic segmentation workflow. Semantic segmentation of cell nuclei (c), conditioned on five broad annotation groups for spots (b). (c,d) Accuracy of cell type composition by semantic segmentation. Fractions of FG pixels in each H&E image (y axis) assigned to each morphological superclass (x axis) in test and train sets (color code) in adult (c, top) and young (d, top) mice, and percent and number (color scale) of pixels with a ground truth superclass label (rows) classified to each label (columns) in in adult (c, bottom) and young (d, bottom) mice. In Box plots: center black line, median; color-coded box, interquartile range; error bars, 1.5x interquartile range; black dots; outliers. Numbers of images used in train and test sets are denoted by “n_train” and “n_test”, respectively, above each subplot. Extended Data Figure 3: Effect of morphological constraints on count-based deconvolution of ST data with NMF. (a) Reconstruction of expression by NMF48 is unaffected by constrained weight parameter (α). Distribution (y axis) of mean squared error (MSE, x axis) between observed (N=11761 genes) and SPOTlight-reconstructed expression (Methods) for N=69,721 spots at different values of α (color code). (b) Cell compositions proposed by NMF decrease in entropy with increased α. Distribution (y axis) of entropy of cell composition vectors predicted by constrained NMF as in (a). Entropy (x axis) is calculated across all 17 snRNA-seq cell types (ksn=17), and thus ranges between 0 (one cell type present) and 1 (all cell types present in equal proportion). (c) Reconstruction of morphological cell compositions is greatly enhanced w [file media-1.pdf]
